# Comment on Richer et al. Night Vision and Carotenoids (NVC): A Randomized Placebo Controlled Clinical Trial on Effects of Carotenoid Supplementation on Night Vision in Older Adults. *Nutrients* 2021, *13*, 3191

**DOI:** 10.3390/nu14132769

**Published:** 2022-07-05

**Authors:** Marina Green-Gomez, Warren Roche, John M. Nolan

**Affiliations:** Nutrition Research Centre Ireland, School of Health Science, Waterford Institute of Technology, X91 K236 Waterford, Ireland; mgreen@wit.ie (M.G.-G.); warren.roche@ucdconnect.ie (W.R.)

Richer and colleagues [1] recently published data in the journal *Nutrients*, claiming that intervention with a carotenoid supplement containing 14 mg of zeaxanthin and 7 mg of lutein (ScreenShieldPro, Eye Promise) over a 6-month period significantly augments macular pigment (MP) and showed “significant improvements in contrast sensitivity with glare… [and] measurable benefits in numerous visual functions that are important for night vision driving…” compared to placebo. As presented in the paper, this trial was funded by Zeavision, the company that sells the ScreenShieldPro carotenoid supplement. Our study of this article strongly disagrees with the results and calls into major question the conclusions made by the authors. Unfortunately, the data presented in the paper are lacking, and we have requested that Richer and colleagues provide us with anonymised sourced data for full analysis. While the data were promised by Richer S., they have not been provided.

We have identified major concerns with their study, summarized below. The main issue which we must highlight relates to study design. The authors claim it is a placebo-controlled trial (RCT), yet the study fails to conduct any between-group analysis [2]. Based on the dependent-samples analysis of the data from the intervention group only, to conclude improvements compared to placebo is a disingenuous claim. By only analysing the experimental group, the effects cannot be justifiably attributed to the intervention or independent of extraneous factors. This represents a major problem and limitation of the study and brings into question the data and conclusions presented in the paper. Furthermore, the methodology of the study is limited with a small and unbalanced sample size (active group *n* = 24 and placebo group *n* = 9). Inclusion criteria were very stringent, potentially creating bias towards an outcome, which is visually evident from the differences between the active and placebo groups at baseline. An assessment of the data provided by the authors clearly shows that the active and placebo groups differ significantly at baseline in terms of MP and visual function variables. Of note, other variables related to MP (i.e., body fat percentage, age, and BMI) are also not comparable, which is a standard expectation for any variable and/or primary outcome measure in an RCT. Moreover, the authors performed no statistical adjustment, which makes it impossible to conclude that between-group differences are due to the intervention.

Macular Pigment

According to the authors, the “right eyes of the supplement group showed an increase in MPOD (mean/SE) 0.35 du/0.04 to 0.41 du/0.05” over a 6-month intervention and claimed statistical significance with *p* < 0.001. However, the device used in the authors’ study (QuantifEye) to measure MPOD exhibits a test–retest variability as high as 0.18 du, which is three times higher than the observed change [3,4]. Therefore, expected variability when using this device is higher than the improvement reported. With the sample size of *n* = 24 in the active group for this study, the standard deviations at baseline and 6-month follow-up can be calculated as approximately 0.196 du and 0.245 du, respectively. Of note, this large measurement variability exhibited by the QuantifEye (referred to in the paper by Richer et al., [1] as the “clinical gold standard”) is comparable to previous reports (see above). Given the mean MPOD values at each time point, this would suggest that the baseline and follow-up results for this group are well-within the observed variability of one another. This can be considered further by analysing the data available in the paper, where the 95% confidence interval (95% CI) of MPOD for the active group at baseline and 6 months are given by (see also our Figure 1):

Baseline 95% CI = 0.35 ± 1.96 × 0.04 = (0.27 to 0.43)

6-month follow-up 95% CI = 0.41 ± 1.96 × 0.05 = (0.31 to 0.51)

In other words, the variance in the system used to measure MP is significantly greater than the change detected in the active group.

Also of note is that the paper fails to provide data for the SE of MPOD for the active group at six months in the left eye, and we find it highly unusual that the authors claim an improvement in MP for one eye (right eye) and not the other (left eye), given the known published interocular symmetry of MP [5,6].

The authors also state that “the mean baseline MPOD of the placebo group was greater than the baseline MPOD of the treatment group” [1]. Given this, the baseline MPOD values between the active and the placebo group are not comparable: the mean MPOD in the right eye is circa 0.58 du in the placebo group and 0.35 du in the active group. This is a difference of circa 66% relative to the Active group. In fact, at six months, the mean MPOD in the placebo group is circa 0.58 du and 0.41 du in the active group, which is a difference of circa 41% relative to the active. The left eye presents similar values, with a difference of circa 66% at baseline and a difference of circa 60% at 6 months. In other words, the placebo group appears to end the experiment with a significantly higher MP than the active group. This is another major limitation of the study, especially given that this difference in baseline MPOD values stated by the authors does not appear to be adjusted in the statistical analysis. Again, a standard practice in this type of study design.

Finally, it is important to highlight that the data presented in the paper for MPOD in the left eye are incorrect. In Figure 1B [1], the active group shows a mean MPOD circa 0.43 du in the left eye at 6 months; however, in the text (4.1. Macular Pigment Optical Density), it is presented as 0.37 du. This error needs to be addressed and corrected. It is difficult to comprehend how this error was missed by the peer-review process.

Visual Function

In terms of visual function, the effects noted within the paper include contrast sensitivity with glare, glare recovery time, useful field of view divided attention, and preferred luminance.

Glare recovery. The description of the glare recovery data in Section 4.3—Glare recovery improvement—[1], notes that “Although there was an improvement in glare recovery time, the placebo group did not change significantly compared to baseline in both eyes, indicating a possible learning curve in testing”. By definition, this implies, by following the same principle, that the improvement in intervention group is also secondary to a learning curve in testing. It is likely, hence, that practice was the primary factor in their improvement. An independent-samples t-test would have demonstrated that there is, in fact, no difference between intervention and placebo groups.

Glare and Contrast improvement. There are no data presented for the placebo group, so we are left to trust that the statistical analysis of change over 6 months in the intervention group is significant, which does not actually matter because practice effects (or other factors) may have contributed to any change in performance. Additionally, there is no figure for these data—the reader is left to wonder what actually happened with regard to the relationship between the placebo and intervention groups. This is a conspicuous omission.

Preferred luminance. As with “Glare recovery” (above), one can easily determine from a visual inspection of Figure 3 [1], that there is no appreciable difference between placebo and intervention groups in terms of change over the trial. In fact, it appears as if the placebo group improved more than the intervention group. As suggested by the authors in Section 4.3 [1], any improvement appears to be due, at least in part, to practice effects. Again, a proper statistical analysis (independent-samples t-test, if data are indeed parametric, which by the sample size is unlikely) of these data would have determined a lack of significant effect.

Useful Field of View, Divided Attention. From a visual inspection of Figure 4b [1], the 3- and 6-month data points for the placebo group are conspicuously high—triple the initial values at baseline and 6 weeks (which showed improvement in line with the intervention group). It is unusual to see reaction times triple over the course of 6 months, especially when they appeared to be consistent during the first 6 weeks. This leaves one to wonder whether, in such a small group (*n* = 9), variability played a role, where a couple of individuals in the group produced outlier-type data at the 3- and 6-month time points. Indeed, upon careful inspection of the figure, one can see very wide error bars associated with the scores for the placebo group at 3 and 6 months. This is not the case for their measures at baseline and 6 weeks. The point here is that response variability may have played a significant role in masking the general pattern of practice effects found throughout the paper—effects that nullify any change that can be attributed to the intervention. Furthermore, the statement in the abstract regarding the divided attention task (data shown in Figure 4b [1]), “the placebo group remain unchanged”, is simply not true. The placebo group changed significantly.

In conclusion, the changes shown in the data are based on within-group analysis of the intervention group only. This is clearly stated by the authors in the text: “the groups were compared longitudinally with themselves and not with each other” [1]. Therefore, any observed change in the intervention group over the course of the trial cannot be attributed to the intervention. This clearly calls into question the title of the paper, which suggests an RCT; this is unfortunately misleading for science, practitioners, and patients. Indeed, based on data presented in the paper, the changes appear to largely be accounted for by-practice and/or time-effects (the placebo group followed a very similar pattern of change). From the analysis conducted by the authors, without between-group analysis, it cannot be concluded that the intervention is better than the placebo. The claim by the authors that the intervention improved MP, multiple aspects of visual function, UFOV scores, and imputed driving crash risk composite scores when compared to a placebo group is incorrect and misleading. Furthermore, there are inconsistencies between the data shown visually and the values used through the text and the statements in the abstract and conclusion. Thus, if patients and healthcare providers are to trust science and we as scientists are to support the peer-review process, it is clear that Richer et al., need to address these major issues. We believe that this report is a gross misrepresentation of the data and strongly recommend its retraction.

## Figures and Tables

**Figure 1:**
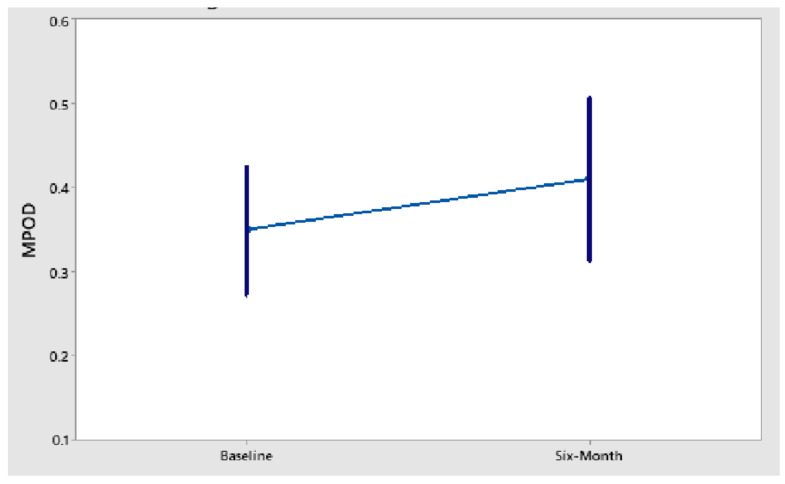
Mean MPOD with 95% confidence interval for baseline and 6-month intervention.
